# Release of Cinnamaldehyde and Thymol from PLA/Tilapia Fish Gelatin-Sodium Alginate Bilayer Films to Liquid and Solid Food Simulants, and Japanese Sea Bass: A Comparative Study

**DOI:** 10.3390/molecules26237140

**Published:** 2021-11-25

**Authors:** Jingwen Chen, Yinxuan Li, Wenzheng Shi, Hui Zheng, Li Wang, Li Li

**Affiliations:** 1 College of Food Science and Technology, Shanghai Ocean University, Shanghai 201306, China; chenjingwenmegan@foxmail.com (J.C.); himavis@foxmail.com (Y.L.); wzshi@shou.edu.cn (W.S.); zheng-h1997@foxmail.com (H.Z.); 2 Engineering Research Center of Food Thermal-Processing Technology, Shanghai Ocean University, Shanghai 201306, China

**Keywords:** biodegradable bilayer film, tilapia fish gelatin, *β*-cyclodextrin inclusion complex, release kinetics, food simulants, active activities

## Abstract

This study aimed to develop an active biodegradable bilayer film and to investigate the release behaviors of active compounds into different food matrices. Cinnamaldehyde (CI) or thymol (Ty) was encapsulated in *β*-cyclodextrin (*β*-CD) to prepare the active *β*-CD inclusion complex (*β*-CD-CI/*β*-CD-Ty). The tilapia fish gelatin-sodium alginate composite (FGSA) containing *β*-CD-CI or *β*-CD-Ty was coated on the surface of PLA film to obtain the active bilayer film. Different food simulants including liquid food simulants (water, 3% acetic acid, 10% ethanol, and 95% ethanol), solid dry food simulant (modified polyphenylene oxide (Tenax TA)), and the real food (Japanese sea bass) were selected to investigate the release behaviors of bilayer films into different food matrixes. The results showed that the prepared *β*-CD inclusion complexes distributed evenly in the cross-linking structure of FGSA and improved the thickness and water contact angle of the bilayer films. Active compounds possessed the lowest release rates in Tenax TA, compared to the release to liquid simulants and sea bass. CI and Ty sustained the release to the sea bass matrix with a similar behavior to the release to 95% ethanol. The bilayer film containing *β*-CD-Ty exhibited stronger active antibacterial and antioxidant activities, probably due to the higher release efficiency of Ty in test mediums.

## 1. Introduction

Active packaging can effectively maintain food quality by controlling microbial growth, enzyme activities, and lipids oxidation, eventually extending the shelf life of the protected foodstuffs [1]. It can be developed by incorporating the antimicrobial and/or antioxidant agents into a polymer matrix or to coat such active compounds on the food contact side of packaging [2]. On account of the natural antioxidant and antimicrobial properties of essential oils (EOs), active packaging films were widely developed by blending straight with EOs [3,4,5]. However, the thermosensitivity of EOs often limits their application in food packaging industrial manufacture such as melt-blending [1,6]. Cyclodextrin molecular inclusion complexation is an encapsulation technology that incorporates unstable and hydrophobic active compounds (guest molecules) into the nontoxic and thermal-resistant cyclodextrins (host molecules) to enhance the solubility and thermostability of guest compounds effectively [7]. *β*-cyclodextrin (*β*-CD) is the most successfully used host molecule because of its low cost, nontoxicity (Generally Recognized as Safe, GRAS), biocompatibility, and effective encapsulation [8]. Hence, active *β*-CD inclusion complexes can be embedded on the only food contact side of packaging materials via a coating to form a bilayer film instead of being incorporated by melt-blending. It overcomes the thermo-volatility of EOs, and the outer layer of bilayer films can be employed as a waterproof layer to decrease the release loss of active substances from the inner coating layer into the outside environment, and it would be easier to maintain an efficient concentration inside the packaging headspace atmosphere over a long time through slow release.

Among active packaging, migratory active packaging has shown great successes in the preservation of foods by releasing active compounds into the food surface [9,10,11,12]. It is thus important to study the release pattern of active compounds from migratory active packaging to the preserved model/real foods as sufficient active compounds should be absorbed into the food matrix to ensure a minimal concentration of the antimicrobial compound is reached to inactivate the microorganisms in model/real foods [13]. However, recent research has mainly focused on investigating the release of active compounds from films into liquid food simulants such as water, ethanol solution, 3% acetic acid solution, and vegetable oil [6,11,14,15,16], whereas studies on the release of the active compounds from films into solid dry food simulants and real foods have rarely been mentioned. Due to the complexity of the food matrix and the changes in food consistency, only using the liquid solution to simulate the release behaviors occurring between active packaging and foods cannot reflect a comprehensive view of the partitioning of active compounds from a food package. Tenax TA, a fine powder of a porous polymer resin based on 2,6-diphenylene oxide that has been recommended in Regulation (EU) No. 10/2011, is a good candidate to be used to study the partitioning of the active agent in a food package containing dry foods [17]. Among the active components in EOs, cinnamaldehyde (CI) and thymol (Ty) were highlighted due to the remarkable antimicrobial activity of cinnamon bark essential oil and oregano essential oil [18,19]. Therefore, relevant application and active activity studies in EVOH-based film and PLA-PCL-based film containing CI and Ty have been investigated [20,21]. However, to the best of our knowledge, there are no studies that have compared the behaviors of CI and Ty releasing from the bilayer packaging films to the food simulants (both liquid and solid food simulants) and real foods (sea bass).

In this study, to develop a novel biodegradable bilayer active film, biodegradable polymer PLA was employed as a waterproof layer, and natural edible biopolymer tilapia fish gelatin-sodium alginate (FGSA) containing *β*-cyclodextrin-cinnamaldehyde (*β*-CD-CI) or *β*-cyclodextrin-thymol inclusion complexes (*β*-CD-Ty) was coated on the surface of the PLA substrate as an active release layer. The current study aimed to compare the release of active compounds (CI and Ty) from the coating layer into liquid food simulants (water, 3% acetic acid, 10% ethanol, and 95% ethanol), solid dry food simulant (Tenax TA), and the real foods (Japanese sea bass fillets). Besides, the active activities and chemical structure, morphological barrier, and mechanical properties of the obtained bilayer films were tested to evaluate their potential as food packaging films.

## 2. Results and Discussion

### 2.1. Quantification of the Active Compounds in β-CD Inclusion Complexes

The results of the quantitative analysis of two active complexes are listed in Table 1. The process efficiency (PE), entrapment efficiency (EE), and drug loading (DL) of *β*-CD-CI were 79.98%, 88.82%, and 11.57%, respectively. *β*-CD-Ty exhibited a poor inclusion performance with a 43.19% lower entrapment of Ty into *β*-CD, compared to the entrapment of CI into *β*-CD. Previous studies have reported comparable entrapment efficiencies of cinnamaldehyde and trans-cinnamaldehyde in *β*-CD inclusion complexes of 91.00% and 84.70%, respectively [22,23]. Many factors from guest molecules, including the molecular weight, molecule volume, molecule polarity, and chemical structure, affect the formation efficiency of inclusion complexes [22].

### 2.2. FTIR Analysis

**Figure 1:**
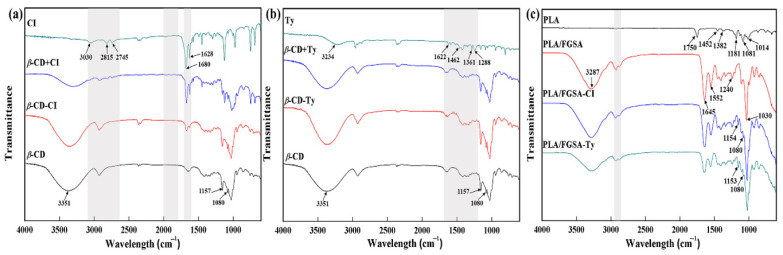
Panel (**a**)**:** FTIR spectra of CI, *β*-CD, their physical mixture (*β*-CD+CI), and inclusion complexes (*β*-CD-CI); panel (**b**): FTIR spectra of Ty, *β*-CD, their physical mixture (*β*-CD+Ty), and inclusion complexes (*β*-CD-Ty); panel (**c**): FTIR spectra of PLA and FGSA-based layers of bilayer films (PLA/FGSA, PLA/FGSA-CI, and PLA/FGSA-Ty).

As shown in Figure 1a,b, the IR spectra of inclusion complexes were almost similar to that of *β*-CD, indicating that the main structures of the two inclusion complexes were the same as that of *β*-CD. Except for both active compounds, all powder samples exhibited a prominent absorption peak at 3351 cm^−1^, corresponding to O−H stretching vibrations mainly originating from the hydroxyl group of *β*-CD (Figure 1a,b) [8]. The intensities of CI characteristic absorption peaks at 1628 cm^−1^ and 1680 cm^−1^ attributed to the stretching vibrations of C=C and C=O groups, respectively, were distinctly decreased in the inclusion complexes (Figure 1a). In addition, peaks of the vibrations of the aromatic ring framework at 2000−1700 cm^−1^ representing a C−H group in the aldehyde group (2745 cm^−1^ and 2815 cm^−1^) and C−H group in the benzene ring (3030 cm^−1^) completely disappeared compared to the physical mixture. These changes suggested that the cavity of *β*-CD prevented the aromatic part of CI molecules from vibrating and, consequently, their original infrared characteristics were hidden. The presence of an hydroxyl group (−OH) in thymol (Ty) was verified by the absorption peaks at 3234 cm^−1^ (Figure 1b). The characteristic peaks of Ty at the 1700–1200 cm^−1^ region of the spectrum corresponded to bands of aromatic C=C stretching vibrations at 1622 cm^−1^, phenyl rings at 1462 cm^−1^, isopropyl groups at 1361 cm^−1^, and C−O stretching vibrations at 1288 cm^−1^ [24,25]. Weak absorption bands at 1622 cm^−1^, 1361 cm^−1^, and 1288 cm^−1^ were recorded in the spectrum of the *β*-CD-Ty inclusion complex, but they could not be found in the physical mixture (*β*-CD+Ty), in line with previous findings [26]. This might be due to the obscured prominent absorption peaks of Ty by very intense and broad *β*-CD bands in the physical mixture and *β*-CD [25]. Moreover, the characteristic absorption peak of the phenyl ring from thymol molecules at 1462 cm^−1^ was sheltered by *β*-CD. All the observations indicated that the inclusion complexes formed between active molecules and *β*-CD.

The chemical structures of the FGSA-based layer and PLA from the obtained bilayer films were characterized by FTIR, as shown in Figure 1c. Typical absorption bands of PLA were recorded in the spectrum: C=O carbonyl stretching (1750 cm^−1^), C−H stretching of −CH_3_ (1452 cm^−1^ and 1382 cm^−1^), C−O stretching (1181 cm^−1^ and 1081 cm^−1^), and −OH bending (1014 cm^−1^) [27,28,29]. The FTIR spectra of all FGSA-based layers showed similar characteristic bands: amide-I (C−O stretching), amide-II (N−H bending), and amide-III (C−N and N−H stretching) with the location of the peaks at approximately 1645 cm^−1^, 1552 cm^−1^, and 1240 cm^−1^ in order, as well as a broadband peak around 3287 cm^−1^ owing to the superposition of the hydroxyl group and the amino group [30,31,32]. However, all intensities of these bands decreased in both PLA/FGSA-CI and PLA/FGSA-Ty groups, indicating that the blend of *β*-CD inclusion complexes reduced the formation of amide bonds from the FGSA composite. Characteristic peaks in the region of 2950–2800 cm^−1^ were related to stretching vibrations of the methylene group [33], and the strong absorption peaks at 1030 cm^−1^ might be related to the interaction induced by the −OH groups of the glycerol and film matrixes [34]. Several extra absorption peaks and bands appearing in the layers with *β*-CD inclusion complexes at 1153 cm^−1^, 1080 cm^−1^, and 800-600 cm^−1^ resulted from the structure of the *β*-CD host molecule.

### 2.3. Morphology of Films

**Figure 2:**
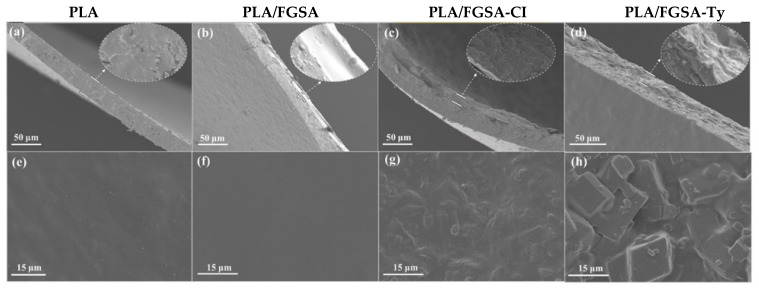
Scanning electron micrographs of cross-section (**a**−**d**) and surface (**e**−**h**) of films (PLA, PLA/FGSA, PLA/FGSA-CI, and PLA/FGSA-Ty).

The dispersion of *β*-CD inclusion complexes in the FGSA composite layer and double-layer structure of bilayer films was observed clearly from the SEM images (Figure 2). The pure PLA film presented a cross-section of a dense and uniform structure, but some tiny protrusions were shown on the surface of the film, being attributed to the process of flowing spread molding. More homogeneous and smooth morphologies of cross-sectional and surface views without cracks and pores were observed from PLA/FGSA (Figure 2b,f), compared to the views of other films, indicating a good adhesion of the FGSA coating on the PLA substrate. *β*-CD complex particles showed a good and compact distribution in the cross-linking structure of fish gelatin and sodium alginate, although their addition caused rough cross-sections and surfaces of the coating layers of PLA/FGSA-CI and PLA/FGSA-Ty (Figure 2c,d,g,h). However, there was an increasing tendency in uniformity and irregularity of the FGSA-Ty coating layer compared to PLA/FGSA-CI, owing to the agglomeration caused by the excessive addition of *β*-CD-Ty complexes (Figure 2d,h). These findings are consistent with previous research that showed that the gelatin film incorporation of functional additives such as fruit peel powder and *β*-CD complexes presented a rougher surface than that of the control group without any addition, with the increase in the amount of the additives [35,36].

### 2.4. Thickness

The significant highest thickness value of 117.40 μm (*p* < 0.05) was observed in the PLA/FGSA-Ty film, followed by the PLA/FGSA-CI film of 114.90 μm. PLA/FGSA and PLA films showed lower thickness values of 99.8 μm and 76.30 μm, respectively. The presence of *β*-CD-CI/*β*-CD-Ty increased the thickness of the films significantly (*p* < 0.05) by more than 15%, compared to the PLA/FGSA film. This increase in the film thickness was also confirmed from the SEM images, in which *β*-CD complex particles were embedded in the FGSA matrix and showed an uneven surface of the coating layer of bilayer films, leading to the increase in the film thickness [30]. A similar effect was observed when plant extract powder, fruit peel powder, and cyclodextrin inclusion complexes were used to prepare the active gelatin-based films [30,33,35]. It was observed that the increase in the concentration of hydroxypropyl-*β*-CD-berberine inclusion complexes increased the thickness of the gelatin film [37].

### 2.5. Water Contact Angle (WCA)

The WCA value can estimate the hydrophilicity or hydrophobicity of the packaging materials surface. A WCA value of more than 90° of the surface is considered hydrophobic, while less than 90° is hydrophilic [35]. Table 2 shows that the WCA value of PLA/FGSA was 85.55° around the threshold of 90° in spite of the hydrophilic nature of gelatin, glycerol, and sodium alginate. This result might be explained by the strong forces that existed between these molecules while being coated on PLA [38]. The WCA values of over 100° of the PLA/FGSA-CI and PLA/FGSA-Ty indicated that the incorporation of complexes improved the surface hydrophobicity of gelatin-based film. The interactions between *β*-CD-Ty/*β*-CD-CI and the polymer matrixes might reduce the hydrophilic groups of the FGSA layer film [39]. Besides, the rough surface of the coating layer with *β*-CD inclusion complexes also increased the WCA value of the films, compared to those films with smooth surfaces [35].

### 2.6. Release of Active Compounds into Food Simulants and Real Foods

#### 2.6.1. Release of Active Compounds into Liquid Food Simulants

**Figure 3:**
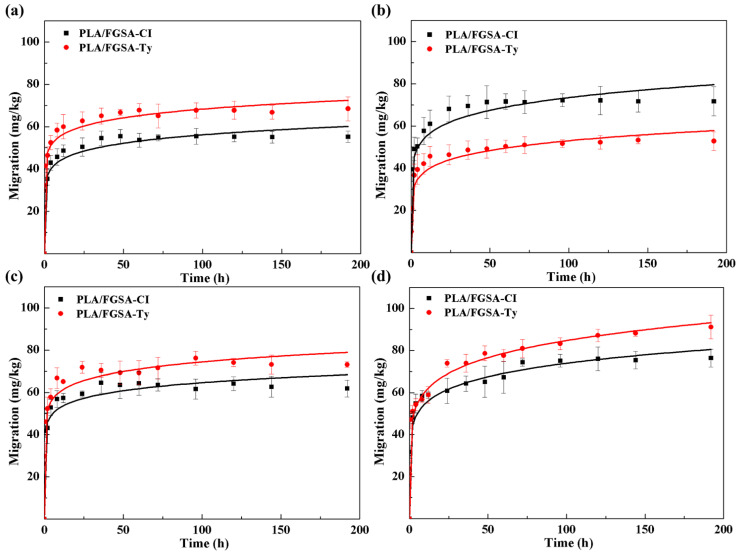
Release of CI and Ty from PLA/FGSA-CI and PLA/FGSA-Ty bilayer films into liquid food simulants: water (**a**); 3% acetic acid (**b**); 10% ethanol (**c**); 95% ethanol (**d**).

The release behaviors of CI and Ty from the FGSA layer in different types of liquid food simulants are shown in Figure 3. The concentrations of CI and Ty in four liquid food simulants initially increased sharply and then reached equilibrium with the slow release (0~196 h, 8 days). It took less than 48 h for both active compounds to reach their equilibrium concentrations in the high-water-content food simulants (water, 3% acetic acid, and 10% ethanol), but longer than 96 h for them to reach the equilibrium in 95% ethanol. The release of CI and Ty from the FGSA network in 95% ethanol solution showed the highest equilibrium concentrations of 76.3 mg/kg and 91.1 mg/kg, respectively, compared to those in water, acetic acid, and 10% ethanol solution. Fick’s second law was used to fit the data of the release behaviors from the bilayer films to the liquid food simulants (Table 3). The model gave a good performance on fitting the release data (R^2^ > 0.9). All *α* values were lower than 0.5, indicating that the release of active compounds into liquid food simulants was quasi-Fick’s diffusion, as suggested by Requena et al. that the release of active compounds is Fick’s diffusion at *α* = 0.5; the release of active compound is considered as quasi-Fick’s diffusion at *α* < 0.05 [40]. The lowest diffusion coefficients (*D*) of active compounds occurred in 3% acetic acid, followed by 95% ethanol, water, and 10% ethanol in order. This suggested that the water content and pH of the food matrix affected the release efficiency of CI and Ty from the bilayer films. Regarding the active compounds, Ty showed higher *D* values than CI in all liquid food simulants except in 3% acetic acid, indicating that those foods with acidic matrixes are not suitable for packaging by PLA/FGSA-Ty.

The release of active compounds from the FGSA layer films into liquid food simulants was accomplished by three processes: diffusion of solvent molecules from simulants to the film-forming matrix; relaxation of FGSA hybrid polymer network; release of active substance (CI/Ty) from *β*-CD cavity into the FGSA-based matrix, and subsequent diffusion from the network structure of the film to the external food simulants matrix [33]. As can be seen, the release efficiency of CI and Ty was affected by the swelling behavior of FGSA coating matrixes, the stability of the guest molecule in the *β*-CD cavity, and the compatibility between active compounds and liquid food simulants. Kaur et al. demonstrated that the release rate of atenolol in the *β*-CD inclusion complex from the hydrogel prepared by chitosan and gelatin decreased in acetic acid solution at a low pH of 2, owing to the fact that the acidic medium can induce more electrostatic repulsions between the chitosan and gelatin polymers, then resulting in a low relaxation rate of the polymer network [41]. Another study observed an easy release of active components (cinnamaldehyde and eugenol) from the inclusion complexes under high humidity conditions of 75% and 100% moisture, because *β*-CD absorbed water molecules and affected the balance of the *β*-CD inclusion complex structure [22]. According to the study of Chen et al., the released amount of citral and trans-cinnamaldehyde from the EVOH-based film into 95% ethanol was 2~3 times lower than the amount released into distilled water due to the high compatibility of active compounds with ethanol solvent [6].

#### 2.6.2. Release of Active Compounds into Tenax TA

**Figure 4:**
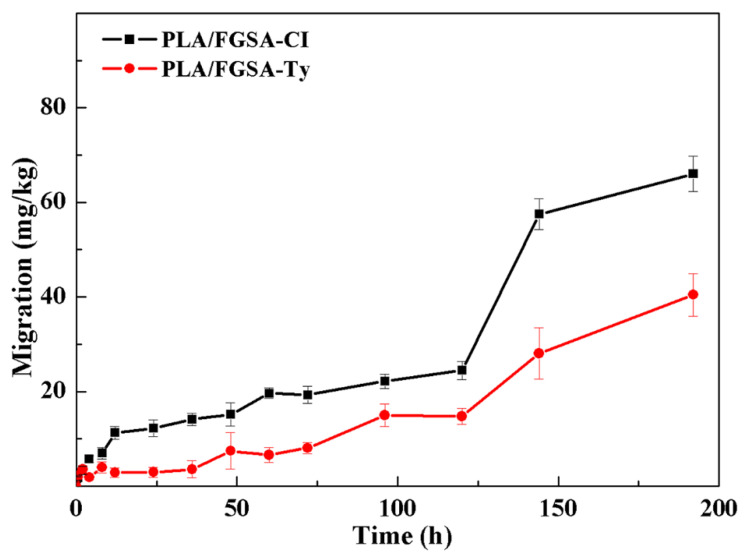
Release of CI and Ty from obtained active bilayer films (PLA/FGSA-CI and PLA/FGSA-Ty) into solid dry food simulant: Tenax TA.

The release pattern of active compounds from PLA/FGSA-CI or PLA/FGSA-Ty film into simulant Tenax TA is given in Figure 4. CI and Ty released slower from the films into Tenax TA than those into liquid food simulants, not reaching equilibrium at the last testing point (196 h). Interestingly, CI released faster than Ty from the bilayer film into Tenax TA with the final concentrations of 66.0 mg/kg and 40.5 mg/kg, respectively. This result means solid dry food is preferable to be packaged by PLA/FGSA-CI. The low release ability of active compounds into solid dry food simulant resulted from the fact that the FGSA complex network and *β*-CD inclusion complex structure were more stable without the swelling induced by the water absorption under the low moisture condition, compared to those in liquid food simulants [42,43]. This result suggested that the developed active bilayer films might be more effective in the preservation of the semi-solid/solid-type foods; the sustained release of active compounds from the film to such a food matrix can ensure they maintain a minimal antimicrobial concentration for a longer time.

#### 2.6.3. Release of Active Compounds into Sea Bass Fillet

**Figure 5:**
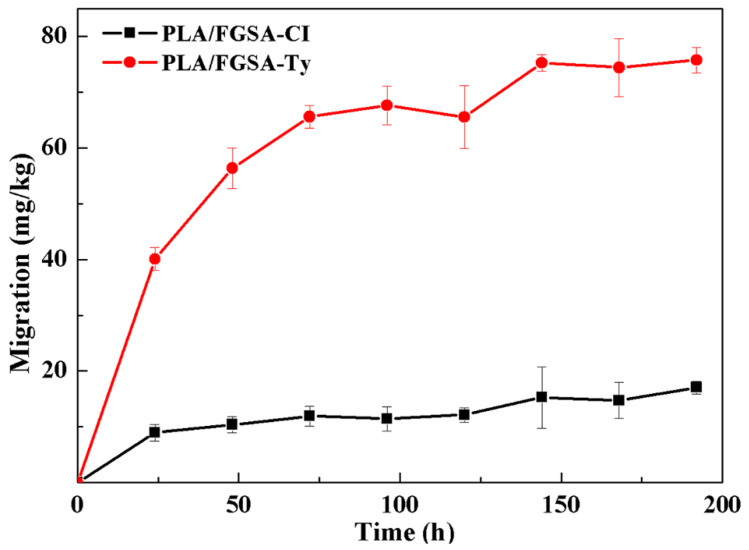
Release of CI and Ty from obtained active bilayer films (PLA/FGSA-CI and PLA/FGSA-Ty) into sea bass fillet.

The release pattern of CI and Ty from the active bilayer film into sea bass fillets at 4 °C during 8 days is shown in Figure 5. The release rates of CI and Ty from bilayer films into sea bass fillets were significantly lower than those into liquid food simulants and higher than those into Tenax TA; however, similar to those nonacidic liquid simulants, Ty was also found at a significantly higher concentration in sea bass fillets than that of CI during the storage (*p* < 0.05). Slow-released CI and Ty tended to approach the state of equilibrium after 144 h, which was comparable to the release activities into 95% ethanol solution. The partitioning of the active compounds in the food package containing fish flesh was more complex than those in the food package containing food simulants as fish flesh is a complex system that simultaneously contains water, protein, fat, and other components. The absorption and migration of CI or Ty into the fish flesh can be affected by the combined factors including the properties of the active components, the composition and (micro)structure of fish flesh matrix, the stability of *β*-CD complex molecules during the partitioning process, and the interaction between the polymer films and the *β*-CD complexes.

### 2.7. Antibacterial and Antioxidant Activities of Films

#### 2.7.1. Antibacterial Activity

**Figure 6:**
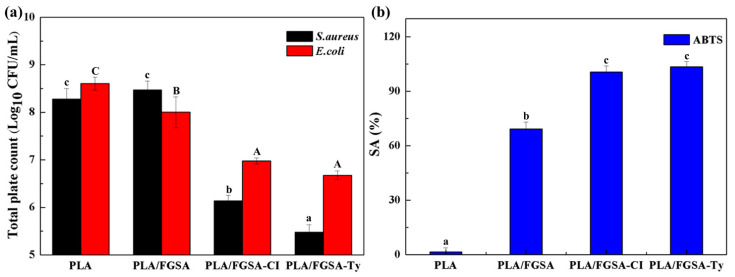
The bacteria populations in different groups were studied and shown at the end of storage (**a**). Antioxidant property of films (PLA, PLA/FGSA, PLA/FGSA-CI, and PLA/FGSA-Ty) for ABTS radical scavenging (**b**). Different subscript lowercase letters and uppercase letters indicate that the means are significantly different (*p* < 0.05).

As shown in Figure 6, both active bilayer films exhibited a significant inhibitory effect on *S. aureus* and *E. coli* (*p* < 0.05); among them, the inhibitory effect against *S. aureus* was stronger than that against *E. coli.* The PLA/FGSA-Ty film was shown to be more effective against *S. aureus* and *E. coli*, compared to the inhibitory effects of PLA/FGSA-CI. The higher inhibition activity against *S. aureus* was attributed to the sensitivity of Gram-positive microorganisms to CI and Ty, supported by previous studies that active film with thymol showed a stronger inhibition in *S. aureus* rather than *E. coli*, and Gram-negative bacteria are more resistant to cinnamon and its constituents including cinnamaldehyde [44,45]. Unlike this study, however, CI possesses a higher antibacterial activity than Ty [46]. This difference can be explained by the fact that the diffusion efficiency of Ty might be higher than CI due to the interactions between the active compound and film, as well as the compatibility between the active compound and solvent when films were immersed in the TSB medium. According to the aforementioned results, Ty released much faster and achieved higher equilibrium concentrations in liquid neutral food simulants. This suggested that the release behavior of active compounds is another key factor that needs to be considered while developing the active food packaging. When applied in food packaging, the CI and Ty sustainedly released from the bilayer films are absorbed on the surface of the food matrix and migrate into the foods during food storage. This releasing process can inhibit the growth of the microorganisms at the places where the interactions between the active compounds and the microorganisms occur. The antibacterial activity of essential oils was primarily achieved by damaging the integrity of the bacterial cell membrane, increasing the permeability of the cell membrane, destabilizing the enzymes located in the cytoderm, and disrupting many cellular activities [1]. Especially, CI was reported to inhibit ATPase and disrupt the outer cell membrane; Ty can affect the permeability of the membrane and induce leakage of intracellular materials by disturbing the lipid fraction of bacterial plasma membranes [47,48].

#### 2.7.2. Antioxidant Activity

ABTS assay was conducted to evaluate the antioxidant property of the PLA/FGSA films. The antioxidants can scavenge the free radicals and chelate metal ions related to the lipid radical chain reaction to slow the lipid peroxidation of food abounded in unsaturated fatty acids. Active bilayer films (PLA/FGSA-CI and PLA/FGSA-Ty) showed almost 100% ABTS radical scavenging activity; especially, the PLA/FGSA-Ty had stronger antioxidant ability. This suggested that the addition of the *β*-CD complexes greatly enhanced the antioxidant capacity of the bilayer films. Interestingly, the control PLA/FGSA film without *β*-CD complexes also exhibited a radical scavenging capacity that reached 69.3%, indicating that the antioxidant effect of the films not only derived from active compounds (CI/Ty) but also from the small molecular polypeptides of fish gelatin protein and sodium alginate [30,49]. The gelatin-based film containing hydroxypropyl-*β*-CD-morin inclusion complexes with a DPPH radical scavenging ability of 92.3% effectively delayed the surface oxidization of freshly cut apple, when the inclusion complex was added at the content of 20% [50]. Another type of fish gelatin film incorporated with *β*-CD-curcumin inclusion complexes also showed an effective antioxidant capacity where ABTS and DPPH free radical scavenging abilities were 93.88% and 73.89%, respectively, at the content of curcumin of about 2.5 mg [36]

## 3. Materials and Methods

### 3.1. Materials and Chemicals

Polylactic acid (PLA, Mark: REVODE 721) was obtained from Zhejiang Hisun Biomaterials Co., Ltd. (Taizhou, Zhejiang, China). *β*-Cyclodextrin (*β*-CD, Mw = 1134.98 g/mol), ethanol (99.8%), and glycerol (≥99.0%) were purchased from Sinopharm Chemical Reagent Co., Ltd. (Shanghai, China). ABTS (2,2′-azino-bis [3-ethylbenzothiazoline-6-sulfuric acid]), cinnamaldehyde (CI, Mw = 132.16 g/mol, 98%), thymol (Ty, Mw = 150.22 g/mol), and their standards were supplied by Shanghai Aladdin Bio-Chem Technology Co., Ltd. Tenax TA (60/80 mesh) and high-performance liquid chromatography (HPLC)-grade acetonitrile were obtained from ANPEL Laboratory Technologies (Shanghai) Inc. Commercial tilapia (*Oreochromis niloticus*) fish gelatin (250 bloom, food grade) was purchased from Vinh Hoan Corporation (Ho Chi Minh, Vietnam). Cultured Japanese sea bass (*Lateolabrax japonicus*) (1000−1500 g in body weight) was supplied alive from a seafood market in Luchao Port town (Shanghai, China). Water was purified by a Milli-Q Plus purification system (Millipore, Shanghai, China).

### 3.2. Preparation of Active Bilayer PLA-FGSA Films

#### 3.2.1. Encapsulation of Active Compounds in *β*-Cyclodextrin

The co-precipitation method was used to prepare *β*-CD molecular inclusion complexes of CI and Ty (*β*-CD-CI, *β*-CD-Ty), according to the procedure described by Chen et al. with some modifications [6].

The UV–Vis spectrophotometry technique was applied for quantitative analysis of the obtained *β*-CD inclusion complexes [51]. The process efficiency (PE), entrapment efficiency (EE), and drug loading (DL) of active complexes were calculated using Equations (1)−(3):

(1) $$\mathrm{PE}=\;\frac{weight\;of\;obtained\;active\;complexes}{weight\;of\;initial\;\beta -CD+weight\;of\;initial\;active\;compounds\;}\times 100\%$$

(2) $$\mathrm{DL}=\frac{weight\;of\;entrapped\;active\;compounds}{weight\;of\;obtained\;active\;complexes}\times 100\%$$

(3) $$\mathrm{DL}=\frac{weight\;of\;entrapped\;active\;compounds}{weight\;of\;obtained\;active\;complexes}\times 100\%$$

where *weight of entrapped active compound* is the active compound (CI or Ty) amount (mg) present in the inclusion complex particles, and *weight of obtained active complexes* indicates the inclusion complex particles amount (mg) finally obtained after vacuum-drying.

#### 3.2.2. Surface Modification of PLA Layer

As a procedure to impart good adhesion of the films, corona discharge is routinely used commercially in factories [52]. Pure PLA film of 75 ± 5 μm was prepared through the melt blending method and then washed with ethanol and deionized water to clean the PLA surface [10]. After drying naturally, PLA films were treated with a laboratory corona treater (MB-200, Nanhui Electrical Equipment Factory, Foshan, China).

#### 3.2.3. Preparation of Tilapia Fish Gelatin-Sodium Alginate Coating Solution

According to the method from the study of Liu et al. [53], the tilapia fish gelatin-sodium alginate composite coating solution (FGSA) was prepared by mixing dissolved 10% tilapia fish gelatin solution (FG) and 2% sodium alginate solution (SA) at 60 °C (FG:SA = 3:2, *v*/*v*). Glycerol (5% of mixture solution, *w*/*w*) was added as a plasticizer into the mixture. For the active FGSA-based coating solution, *β*-CD inclusion complexes containing 1% equivalent weight of active compounds (either CI or Ty) were added and then dispersed evenly by a FJ200-SH homogenizer (Shanghai Specimen and Model Factory, Shanghai, China) in the FGSA composite solution system for 5 min at a speed of 8000 r/min. Finally, the coating solutions (FGSA, FGSA-CI, and FGSA-Ty) were degassed by using the sonicator (120 W) for 5 min.

#### 3.2.4. Coating Process

The same amount of resulting coating solution equaling the mass of the PLA substrate films was spread evenly to control the film thickness, and it was then followed by drying at 50 °C in a constant-temperature drying oven for 30 min. Then, the whole coating process mentioned above was repeated once more. In total, 3 types of bilayer films were obtained: PLA/FGSA (PLA substrate films coated only by FGSA composite solution as controls), PLA/FGSA-CI, and PLA/FGSA-Ty (PLA substrate films coated by FGSA composite solution containing active complexes *β*-CD-CI or *β*-CD-Ty).

### 3.3. Fourier-Transform Infrared Spectroscopy (FTIR) and Scanning Electron Microscopy (SEM)

Test samples of CI, Ty, *β*-CD, physical mixtures of *β*-CD and CI/Ty, and inclusion complexes were prepared by grounding with dry potassium bromide (KBr) at a ratio of 1:100 (*w*/*w*). IR spectra from 4000 to 600 cm^−1^ (mid infrared region) of KBr samples and the FGSA-based layer of bilayer films were measured using a Nicolet Nexus Avater 370 FTIR spectrophotometer (Thermo Nicolet Corporation, Madison, WI, USA). All analyses were recorded at a resolution of 4 cm^−1^ and expressed as the average absorbance value of 32 scans.

The micromorphology of films was observed by SEM (Hitachi SU5000, Tokyo, Japan). Samples were placed on a conductive double-faced adhesive tape and sputter-coated with a gold coating at 15 mA for 60 s to increase their conductivity. Finally, the observation was performed at an acceleration voltage of 5 kV.

### 3.4. Thickness

A spiral micrometer (Guanglu Measuring Instrument Co., Ltd., Guilin, China) was used to measure the thickness of film samples at five random positions around the film, after the measurement, and all films were stored in a moisture-proof aluminum foil bag with food-grade silica gel as a desiccant.

### 3.5. Water Contact Angle (WCA)

The surface hydrophobicity of films was determined through the water contact angle (WCA) parameter according to the method described by Huang et al. [27]. A droplet of distilled water (3 μL) was carefully dropped on the film surface using a microsyringe, and then the contact angles and images were recorded immediately by a JC2000c video-based contact angle meter (Shanghai Zhongchen Digital Technic Apparatus co., Ltd., Shanghai, China)

### 3.6. Release of Active Compounds into Food Simulants and Real Food

#### 3.6.1. Release Test of Active Compounds into Liquid Food Simulants

According to the method described by Yang et al. [54], in brief, film strips were completely immersed into the four liquid food simulants mentioned above at a ratio of 6 dm^2^/L, respectively, and the immersion solutions were sealed and stored in the dark at 4 °C. Then, each simulant of 1 mL was sampled at definite time intervals. Samples were filtered through a 0.22 μm nylon membrane filter, and HPLC analysis was performed to measure the concentration of CI and Ty in simulants.

#### 3.6.2. Mathematical Models of Liquid Food Simulants

To confirm whether the release process of active compounds comply with the commonly used Fick’s second law, the following equation can be applied to fit the release occurring in liquid food simulants [15]:

(4) $$\frac{M_{F,t}}{M_{F,\;\infty }}=kt^{\alpha }$$

where *M_F,t_* indicates the release concentration of active compounds in food simulants at a particular time t (s), *M_F,∞_* indicates the maximum release concentration of active compounds in the simulant while achieving equilibrium, *k* is the rate constant incorporating characteristics of the matrix during the diffusion process, and *α* is the diffusional exponent providing information about the dynamics involved in the release process.

Based on Fick’s second law, Equation (5) was applied to study the diffusion kinetics [55].

(5) $$\frac{M_{F,t}}{M_{F,\;\infty }}=1-\sum_{n=0}^{\infty }\frac{8}{{\left(2n+1\right)}^{2}\pi^{2}}\exp\left[-\frac{{\left(2n+1\right)}^{2}\pi^{2}}{L_{p}^{2}}Dt\right]$$

where *D* (m^2^ s^−1^) is the diffusion coefficient of active films, *t* (s) is time, and *Lp* (m) is the thickness of the FGSA layer film. The initial diffuse period of migration can be simplified into Equation (6), and then the coefficient *D* was calculated from the slope of the plot of *M_F,t_*/*M_F,∞_* as a function of *t*^1/2^ [6,55]:

(6) $$\frac{M_{F,t}}{M_{F,\;\infty }}=\frac{2}{L_{p}}{\left(\frac{Dt}{\pi }\right)}^{\frac{1}{2}}\;$$

#### 3.6.3. Release Test of Active Compounds into Solid Dry Food Simulant

To simulate the release of active compounds from the bilayer films into the solid dry foods, 0.416 g of Tenax TA was placed evenly on the bottom of the 100 mL screw-cap glass container and was placed in contact with the coating layer side of bilayer films (0.1 dm^2^) directly. Then, the containers were sealed and stored in the dark at 4 °C. Periodically, Tenax TA was extracted with 5 mL of acetonitrile using an ultra-sonicator for 5 min, then the supernatant was filtered through a 0.22 μm nylon membrane filter membrane. Finally, HPLC was used to quantify the concentration of CI and Ty.

#### 3.6.4. Release Test of Active Compounds into Sea Bass Fillets

An eight-day release test of CI and Ty was studied, and sea bass fillets of 25.0 g packaged by PLA/FGSA-CI and PLA/FGSA-Ty films (2.34 dm^2^) were stored at 4 °C and sampled every 24 h. Active compounds in sea bass fillets were recovered by the extraction of acetonitrile; then, the rotary evaporation of obtained acetonitrile extracts was performed to increase the concentration of target substances [54]. HPLC detection was conducted after the extraction process.

#### 3.6.5. HPLC Analysis

The concentrations of CI and Ty were quantified using an Agilent HPLC 1260 equipped with a G4212B diode array detector (Agilent 1260, Agilent, Santa Clara, CA, USA) and an Agilent ZORBAX SB-C18 analytical column (4.6 mm × 250 mm, 5 μm). CI and Ty standard calibration curves were prepared using acetonitrile as a solvent, and the diluted standard concentration ranges were 0.5−200 μg/mL and 5-250 μg/mL, respectively. The mobile phase was a mixture of Milli-Q water and acetonitrile (3:2, *v*/*v*) with a flow rate of 1 mL/min, the column temperature was 30 °C, and the analysis time was 10 min.

### 3.7. Antibacterial and Antioxidant Activities of Films

Two bacterial strains, *Escherichia coli* ATCC 8099 and *Staphylococcus aureus* ATCC 6538, were used as the test microorganisms. The antimicrobial activities of the developed films were examined according to Ma et al. with some modifications [11]. Sterilized film pieces of 0.5 g were placed in tubes containing 10 mL of Tryptone Soy Broth (TSB) and then stored in a refrigerator at 4 °C for 5 days. Each strain was subcultured in TSB at 37 °C until the exponential phase for use. A total of 100 μL of exponential phase bacteria (colony count of 10^5^ CFU/mL) was inoculated into tubes with 10 mL of TSB medium and shaken at 200 rpm for 12 h. The diluted suspensions were spread on plates with tryptic soy agar (TSA) medium. Colonies were counted after incubating at 37 °C for 24 h.

The antioxidant activity of the films was determined by measuring the ABTS free radical scavenging ability from the film soak solution. Film samples were cut into pieces and soaked in 95% ethanol at a concentration of 6 dm^2^/L. After 4 h of immersion at 60 °C, fresh ABTS working solution was mixed with film soak solution or 95% ethanol as blank at a ratio of 4:1 (*v*:*v*), and then kept for 6 min before being measured with the spectrophotometer at 734 nm. The fresh ABTS working solution was prepared following the method of Hanani et al. [35]. The ABTS radical scavenging activity (SA) was calculated according to following formula:

(7) $$\mathrm{SA}/\%=\frac{A_{0}-A}{A_{0}}\times 100\%$$

where A_0_ is the absorbance value of blank and A is absorbance value of samples.

### 3.8. Statistical Analyses

All the experiments were performed in triplicate. Data were presented as means ± standard deviation (SD). SPSS version 20.0 software was used for statistical analysis. Data were assessed with one-way analysis of variance (ANOVA) by using Duncan’s multiple range test. A value of *p* < 0.05 was considered significant.

## 4. Conclusions

CI or Ty was encapsulated into *β*-CD inclusion complexes with the drug loading of 11.57% or 9.65%, respectively; and they were added as functional substances into the FGSA layer of PLA-based bilayer films to prepare the biodegradable active food packaging films. Incorporation of the *β*-CD inclusion complexes into the FGSA matrix increased the thickness, WVP, and WCA values significantly, markedly decreased the OTR value, and influenced TS and EAB values of bilayer films. The water content and pH of food matrixes affected the release of active bilayer films, which can be reflected by the phenomenon that weaker release behaviors of active compounds from bilayer films were found in low-moisture and acidic food simulants, while the weakest release behavior was found in Tenax TA, compared to other systems. The releases of CI and Ty into sea bass fillets were lower than those in the liquid food simulants, though slightly closer to those in 95% ethanol. The antimicrobial and antioxidant activities of the bilayer films were related closely to their active compound release abilities; both developed bilayer films, especially the PLA/FGSA-Ty, significantly inhibited the growth of foodborne pathogens (*S. aureus* and *E. coli*) and scavenged the ABTS free radical cations (*p* < 0.05). Our results highlight the importance of considering the release behavior of active compounds in active food package-containing foods with different matrix consistencies (e.g., liquid or solid foods, neutral or acidic), while applying such a type of active packaging. The developed bilayer packaging films show great potential to preserve the (semi-)solid food products due to the prolonged release of the active compounds and their desired antimicrobial and antioxidant properties.

## Figures and Tables

**Table 1:** Process efficiency, entrapment efficiency, and drug loading of active *β*-CD inclusion complexes.

*β*-CD Inclusion Complexes	Process Efficiency (*w*/*w* %)	Entrapment Efficiency *(w*/*w* %)	Drug Loading (*w*/*w* %)
*β*-CD-CI	79.98 ± 0.04 ^b^	88.82 ± 0.04 ^b^	11.57 ± 0.00 ^b^
*β*-CD-Ty	62.35 ± 0.01 ^a^	50.46 ± 0.01 ^a^	9.65 ± 0.09 ^a^

Data are presented as means ± standard deviation. Means followed by a different superscript letter in the same column are significantly different (*p* < 0.05).

**Table 2:** Water contact angle of films (PLA, PLA/FGSA, PLA/FGSA-CI, and PLA/FGSA-Ty).

Film Type	WCA (°)	Image
**PLA**	71.90 ± 1.55 ^a^	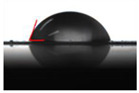
**PLA/FGSA**	85.55 ± 2.88 ^b^	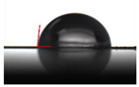
**PLA/FGSA-CI**	106.74 ± 6.35 ^c^	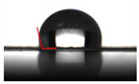
**PLA/FGSA-Ty**	108.21 ± 6.43 ^c^	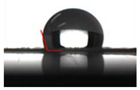

Data are presented as means ± standard deviation. Means followed by the different superscript letter in the same column are significantly different (*p* < 0.05).

**Table 3:** Diffusivity and diffusional exponent of CI and Ty in obtained active bilayer films in contact with different liquid food simulants.

Active Compounds	Simulants	*α*	*k*	R^2^	*D* (m^2^ s^−1^)
CI	water	0.11	0.26	0.96	2.16 × 10^−13^
	3% acetic acid	0.12	0.22	0.92	1.77 × 10^−13^
	10% ethanol	0.087	0.34	0.93	2.96 × 10^−13^
	95% ethanol	0.13	0.18	0.96	1.88 × 10^−13^
Ty	water	0.090	0.32	0.97	3.31 × 10^−13^
	3% acetic acid	0.13	0.19	0.91	1.05 × 10^−13^
	10% ethanol	0.091	0.32	0.94	3.61 × 10^−13^
	95% ethanol	0.15	0.15	0.96	2.67 × 10^−13^

Obtained by fitting the data from Figure 3 with Equation (6).

## Data Availability

The data presented in the study are available upon request from the corresponding authors.

## Sample Availability

Samples of the compounds used for experiment including *β*-CD, ethanol, glycerol, ABTS, cinnamaldehyde, thymol, Tenax TA (60/80 mesh) and HPLC-grade acetonitrile are available from the authors.
